# Sample size in a prognostic study: prep (prediction of risks in early onset pre-eclampsia)

**DOI:** 10.1186/1745-6215-14-S1-P49

**Published:** 2013-11-29

**Authors:** John Allotey, Julie Dodds, Sally Kerry, Shakila Thangaratinam

**Affiliations:** 1 Queen Mary University of London, London, UK

## Background

Significant numbers of studies struggle to recruit to target and request extension of time or additional funding. PREP is a study to develop a prediction model for adverse maternal and fetal outcomes in women with early onset pre-eclampsia. Pre-eclampsia is a condition in pregnancy associated with raised blood pressure and proteinuria and is one of the main causes of maternal and fetal mortality and morbidity. Accurate prediction of risks will aid clinicians in planning appropriate management.

## Sample size

In developing our prediction model, we aimed to examine 10 candidate predictor variables for inclusion. Simulation studies examining predictor variables for inclusion in logistic regression models suggest 10 events are necessary for each predictor, to avoid overfitting. We therefore require at least 100 women with an event. From our systematic reviews, 20% of women with early onset pre-eclampsia were expected to have adverse maternal outcomes. PREP plans to recruit 500 women with early onset pre-eclampsia.

## Recruitment

After an initial period of slow recruitment, rates increased. From 47 sites, 363 women have been recruited against the 357 target to date.

## Sample size determined by event rates

Interim analysis of observed study outcomes identified rate of outcomes to be less than anticipated 20%. After discussion with the joint DMC/SSC, the decision was made to increase the sample size until 100 events are obtained.

## Conclusion

Sufficient numbers of outcomes are necessary to develop a robust multivariate prediction model for given number of variables. Investigators need to take this into consideration during design, analysis and interpretation.

